# Announcing the 2017 *Medicines* Travel Award for PostDocs

**DOI:** 10.3390/medicines4010004

**Published:** 2017-01-24

**Authors:** Gerhard Litscher

**Affiliations:** Research Unit for Complementary and Integrative Laser Medicine, Research Unit of Biomedical Engineering in Anesthesia and Intensive Care Medicine, and TCM Research Center Graz, Medical University of Graz, Auenbruggerplatz 29, 8036 Graz, Austria; gerhard.litscher@medunigraz.at; Tel.: +43-316-385-13907; Fax: +43-316-385-13908

For the *Medicines* Travel Award 2017*,* we received in total 72 applications from all over the world, most of which were of a very high quality. A decision has been reached by four experts from four different continents (Asia, Australia, Europe and America) and by the editor-in-chief of *Medicines*.

As Editor-in-Chief of *Medicines*, I am pleased to announce the winner of the *Medicines* Travel Award for 2017. The travel award was granted to Dr. Peggy M.P.C. Bosch, a postdoctoral research associate at Radboud University, Nijmegen, The Netherlands, working on acupuncture in the treatment of schizophrenia, sleep and depression. The award consists of 800 Swiss Francs to attend any academic conference during 2017.

**Figure:**
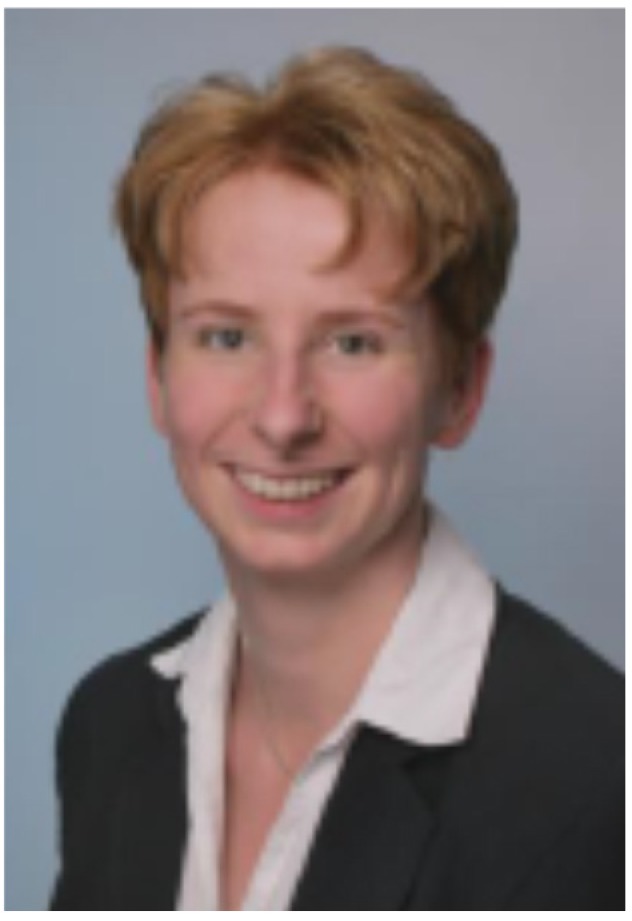
**Dr. Peggy M.P.C. Bosch**

Dr. Peggy Bosch holds a master’s degree in Clinical Psychology from Radboud University, Nijmegen, The Netherlands. After receiving her master’s degree, she worked as a psychologist in a forensic psychiatric department, in an addiction department, in an outpatient clinic, and in a trauma department, all at LVR-Klinik Bedburg-Hau in Germany. She worked as an unpaid PhD-student, alongside her clinical psychological work, on a project on acupuncture in the treatment of schizophrenia, sleep and depression and received her PhD-degree from Donders Institute for Brain, Cognition, and Behaviour, Centre for Cognition, Radboud University Nijmegen in 2015. She is currently continuing her research in this direction (as an unpaid postdoc) at both Radboud University and at LVR-Klinik Bedburg-Hau. Dr. Bosch is a reviewer for several journals, was chair at several conferences, and often functions as (keynote) speaker or lecturer. She cooperates with Kyung Hee University, Seoul, Republic of Korea, as well as with Harvard University, Cambridge MA, USA and has published in high impact factor journals, such as *Science*, *The Lancet*, *Annals of Internal Medicine*, etc. Her research focus still lies on acupuncture in psychiatry, although she has also co-authored publications on other themes: Parkinson’s disease, dementia, or methodology of acupuncture research. Recent work, together with colleagues from Kyung Hee University and Harvard University, focused on differences in the neural and psychophysical responses to acupuncture between males and females and also on the differences in responses to acupuncture between left and right sided acupuncture stimulations, using the functional Magnetic Resonance Imaging technique. However, since a lack of research funding has always been a problem for her acupuncture research during all those years, with the support of the *Medicines* Travel Award, she will now be able to present her work at a highly influential conference this year.

Congratulations to Dr. Peggy Bosch on behalf of the jury and the *Medicines* editorial and publishing teams! 

